# Research on the Reproduction of *Trichogramma chilonis* Based on *Samia cynthia ricini* Eggs: Temperature, Functional Response and Proportional Effect

**DOI:** 10.3390/insects15120963

**Published:** 2024-12-03

**Authors:** Xi Yuan, Dunsong Li, Weili Deng

**Affiliations:** Institute of Plant Protection, Guangdong Academy of Agricultural Sciences, Key Laboratory of Green Prevention and Control on Fruits and Vegetables in South China Ministry of Agriculture and Rural Affairs, Guangdong Provincial Key Laboratory of High Technology for Plant Protection, Guangzhou 510640, China; 13427690102@163.com (X.Y.); 13822169731@163.com (W.D.)

**Keywords:** parasitism rate, emergence rate, wasp-to-egg ratios, propagation efficiency

## Abstract

*T. chilonis* is a commonly used biological agent, but its existing host eggs have shown some problems in the breeding of *T. chilonis*, and the search for more suitable host eggs is imminent. Here, we focused on *S. c. ricini*, an intermediate host that was used in the past for *Trichogramma* spp. but has since received less attention. We attempted to understand the effects of developmental temperature and duration on its longevity and egg production, evaluated the functional response of *T. chilonis* to *S. c. ricini* egg, and screened for a suitable wasp-to-egg ratio for the production of *T. chilonis*. Our results showed that the developmental temperature and duration after the emergence of *T. chilonis* significantly affected adult longevity and oviposition, that 25–28 °C is a suitable temperature range for the survival and e-g laying of *S. c. ricini*, and that a developmental duration of at least 24 h was required to obtain more qualified *S. c. ricini* eggs. *T. chilonis* demonstrated a type II functional response to *S. c. ricini* eggs; different wasp-to-egg ratios significantly affected the propagation efficiency of *T. chilonis* reproduction from *S. c. ricini* eggs, and the best reproduction efficiency was achieved with wasp-to-egg ratios of 1:2 and 1:3, Considering that there were no significant differences in most parameters between the two treatments, as well as cost concerns, we concluded that wasp-to-egg ratios of 1:2 are an important parameter that could be applied. Our results may provide some valuable insights into the mass rearing of *T. chilonis*.

## Simple Summary

*Trichogramma chilonis* is widely used for biological control, but there is a lack of efficient and available host eggs for the production of this parasitoid. *Samia cynthia ricini*, an economically valuable, non-mulberry silkworm, was previously reported to be used for the reproduction of *Trichogramma* spp. but subsequently received less attention, and more details about its use are unknown. Through laboratory experiments, we found that the suitable developmental temperature for adult *S. c. ricini* is 25–28 °C, and the adults must undergo at least 24 h of development time after emergence to lay more qualified eggs. *T*. *chilonis* has a type II functional response to *S. c. ricini* eggs. In brief, this means that as *S. c. ricini* egg density increases, the number of eggs parasitized by *T*. *chilonis* will gradually increase, but the growth rate will slow down and eventually reach a saturation state. Accordingly, by coordinating the ratio of parasitoid wasps to host eggs, we found that *S. c. ricini* eggs demonstrated excellent reproductive efficiency in terms of the reproduction of *T. chilonis* when the ratios were 1:2 and 1:3. We concluded that *S. c. ricini* eggs are potentially an excellent host egg for breeding *T*. *chilonis* and should be given more attention.

## 1. Introduction

Biological control through the release of egg parasitoids is considered to be a safe and sustainable approach to achieve the effective management of agricultural pests [1]. *Trichogramma* species are the most widely exploited and used for pest management worldwide [2], and large-scale field releases for maize pest control have been carried out in China, with significant ecological and economic benefits [3]. *Trichogramma chilonis* (Ishii) is a *Trichogramma* spp. with high reproductive capacity [4], and has gained attention in recent years as a potential biological agent in the management of many important agricultural pests [5,6,7]. However, the achievement of the large-scale release of egg parasitoids is closely related to the cost of rearing [8], and exploring techniques and methods that can enable the mass rearing of *T*. *chilonis* is a critical step towards the use of them as a biological agent for successful biocontrol practices.

*Trichogramma* spp. spend all periods of their lives in host eggs except for their adult lives, so obtaining suitable intermediate host eggs is crucial for the mass production of *T*. *chilonis* [9]. Currently, the intermediate hosts used for the mass reproduction of *Trichogramma* spp. in China are the eggs of *Corcyra cephalonica* (Stainton) [10,11], *Sitotroga cerealella* (Olivier) [12,13], *Samia cynthia ricini* Boisduval [14] and *Antheraea pernyi* silk [11,15]. Among them, *C*. *cephalonica* and *S. cerealella* are widely used as intermediate hosts due to their ease of feeding, wide range of possible forage sources, and ability to reproduce indoors throughout the year and facilitate mass production [9,16,17]. However, the small egg sizes of *C*. *cephalonica* (0.5–0.9 mm) [18] and *S. cerealella* (0.5–0.6 mm) [17] resulted in inefficient wasp reproduction because only one *Trichogramma* individual can complete its development per insect egg. Moreover, the viability of *Trichogramma* spp. decreased after reproduction with *S. cerealella* eggs and parasitized eggs do not tolerate cold storage [19,20]; therefore, the use of small host eggs for the mass production of *Trichogramma* spp. is uneconomical [21]. In contrast, *A. pernyi* eggs are the most successful host eggs in the mass rearing of *Trichogramma* spp., with the advantages of a high reproduction rate, convenient transport and low cost [22]. Moreover, compared with the traditional reproduction using *C*. *cephalonica* eggs, the body size, longevity and lifetime fecundity of *Trichogramma ostriniae* Pang et Chen are superior when using *A. pernyi* eggs as a host, which make them a more effective natural enemy for biocontrol [23]. As early as 1962, Lin et al. [24] found that *T. chilonis* could be reproduced using *A. pernyi* eggs, carried out biological observations of *T. chilonis* parasitized in *A. pernyi* eggs and found that *A. pernyi* eggs had high efficiency in terms of *T. chilonis* reproduction. However, the production of *A. pernyi* is dependent on the use of *Quercus palustris* Münchh. in north-eastern China, and climatic and geographic differences have resulted in there being no cost advantage for the use of *A. pernyi* for the development of *T. chilonis* in southern China [22]. In addition, a recent study found that the parasitism and emergence rates of *T. chilonis* were significantly reduced when the eggs of *A. pernyi* were used as an alternative host, seriously affecting the propagation quality of *T. chilonis* and the field control effects [14]. Therefore, there is an urgent need to find a more cost-effective and high-efficiency intermediate host for the better reproduction of *T. chilonis*.

*S. c*. *ricini* (Lepidoptera: Saturniidae), is one of the most highly developed, domesticated and commercially available non-mulberry silkworms of economic importance [25]. This silkworm feeds on several host plants but mainly on *Ricinus communis* L. leaves [26,27] and can be raised indoors and reproduced for several generations per year [25]. In 1956, Pu et al. [19] used *S. c*. *ricini* eggs to breed *T. chilonis*, and found that each egg of *S. c*. *ricini* could breed about 28 *T. chilonis* offspring, the female ratio of the offspring was more than 80% and the propagation coefficient was 13.0 to 22.6 fold, which proved that the *S. c*. *ricini* eggs were an excellent host for *T. chilonis*. Since then, Liu et al. [28,29] have overcome the problem of raising *S. c*. *ricini* on artificial feed, and successfully achieved the annual breeding of *S. c*. *ricini*. *T. chilonis* reared from the eggs of *S. c*. *ricini* were then successfully released into the field [30,31]. However, in the early 1980s, due to the reform of China’s rural economic system and for other reasons, *S. c*. *ricini* was no longer reared at a large scale, and it ceased to be used in China as a host for the mass rearing of *Trichogramma* spp. [3]. In recent years, *S. c*. *ricini* has received renewed attention due to the continued high price of *A. pernyi* eggs [32] and the limited number of species of *Trichogramma* parasitoids that can be reproduced by *A. pernyi* eggs [33], making it necessary to improve our understanding of the usage of *S. c*. *ricini* eggs for the reproduction of *T. chilonis*. However, before we carry out the above-mentioned studies, we should focus on several key parameters of *S. c. ricini* rearing, such as temperature, light and the use of artificial feeds. Although previous studies have obtained important results regarding the effects of temperature on the development and reproduction of *S. c. ricini* [34] and the exploitation of artificial feeds [29], most of these studies concentrated on the pre-adult stage. In contrast, little research has dealt with the impact factors after the eclosion of insects, such as developmental temperature and duration, which significantly affect the quality of subsequent egg-laying [35]. Therefore, to better understand the factors affecting the reproduction of *S. c. ricini*, it is essential to expand our knowledge of the factors affecting the stage after eclosion, which will help to improve the efficiency of rearing and the quality of egg production for *S. c. ricini*.

The functional response refers to the correlation between the predation rate of a single predator and the different densities of its prey within a specific period [36,37], and is an important indicator when describing the dynamic relationship between the natural enemies of pests and the population quantity of pests and evaluating the parasitism or predation efficiency of natural enemies on pests [38,39]. Functional responses are typically distinguished into three response types, which are characterized by the number of prey consumed, which increases linearly (type I), hyperbolically (type II) or sigmoidally (type III) [36]. Understanding the functional responses of *Trichogramma* spp. can help to predict the pest control potential of *Trichogramma* spp. at different pest densities, which helps to formulate a more scientific and rational biological control strategy to determine the appropriate release number of *Trichogramma* spp. for optimal pest control and to improve the success and efficiency of biological control. For example, the handling time and attack rate of *Trichogramma euproctidis* (Girault) on *Helicoverpa armigera* (Hübner) eggs were 0.6898 h and 0.00823 h, respectively, and the maximum daily parasitism was 34.79 eggs per wasp [40], whereas the maximum number of parasitized eggs by each female of *Trichogramma achaeae* Nagaraja and Nagarkatti and *T. chilonis* on *Tuta absoluta* (Meyrick) was 16.23 and 11.05 eggs in 24 h, respectively [41]. Therefore, knowledge of the functional responses of *Trichogramma* spp. to different pests is of great practical importance for the development of control strategies and breeding strategies. However, to our knowledge, little of the literature has reported the functional responses of *T. chilonis* to the eggs of *Spodoptera exigua* Hübner and *Plutella xylostella* Linnaeus [41], *C*. *cephalonica* [7], *T. absoluta* [42], *Galleria mellonella* Linnaeus and *Chilo sacchariphagus* Bojer [43]. The potential of *S. c*. *ricini* as intermediate hosts for the mass production of *T. chilonis* and the functional response of *T. chilonis* to castor *S. c*. *ricini* eggs remains unclear. Based on this, this study intends to (1) explore the impacts of developmental temperature and duration on the oviposition of *S. c*. *ricini*; (2) reveal the functional response of *T. chilonis* to the eggs of *S. c*. *ricini*; (3) elucidate the effects of different ratios of *T. chilonis* to *S. c. ricini* eggs (wasp-to-egg ratios) on the reproduction of *T. chilonis*. This study conducts a preliminary exploration on the methods of reproduction of *T. chilonis* with *S. c*. *ricini* eggs, which may provide a reference for the industrialized production of *T. chilonis* in the future.

## 2. Materials and Methods

### 2.1. Insect Sources and Feeding

*S. c*. *ricini* eggs were provided by the Institute of Plant Protection, Guangxi Academy of Agricultural Sciences, and *S. c. ricini* were reared in an artificial climate chamber (RDZ-300D-4W, Ningbo Jiangnan Instrument Factory, Ningbo, China) with castor leaves for experimental use. The culture temperature was 25 ± 1 °C, relative humidity (RH) was 75 ± 10%, L:D = 14:10 (the following culture conditions are the same as this, unless otherwise stated), fresh castor leaves were added and food scraps were cleaned up daily.

Pupae of *T*. *chilonis* were collected from the natural egg masses of *C. sacchariphagus* and *Chilo infuscatellus* (Snellen) in the sugarcane field at Nanning City, Guangxi Zhuang, Autonomous Region. After identification and purification indoors, *T. chilonis* were bred in an artificial climate chamber. *T. chilonis* was used in the experiment after breeding for several generations with eggs of *C*. *cephalonica* and sufficient eggs were given at once to *T. chilonis* for parasitism during propagation.

### 2.2. Experimental Design

*S. c. ricini* that cocooned on the same day were selected and after 10 days of normal development at room temperature, the cocoon shells were carefully cut open with scissors and the pupae of *S. c. ricini* were cautiously taken out. After the sex identification of pupae under a dissecting microscope, female and male pupae were placed in separate containers to develop individually, to ensure that female moths that fledged were unmated prior to the experiment and that there was no food supplementation during the culture period.

#### 2.2.1. Developmental Temperature

Female pupae of *S. c. ricini* with a similar size and weight were selected and each pupa was individually packed in a cup and sealed with gauze mesh. After the pupae emerged, the unmated female moths that emerged on the same day were selected and packed into separate cups that were sealed with gauze mesh. Four treatments at different temperatures were set at 22, 25, 28 and 31 °C. The relative humidity was 75 ± 10% and the photoperiod was 14L:10D. Each treatment contained 30 female moths and was repeated three times.

During the adult stage, the number of eggs laid and the survival of each adult moth were observed daily. After the female moth died, the number of qualified eggs remaining in each moth was investigated via dissection, and the day-by-day average number of self-laying eggs, self-laying eggs per female, eggs remaining per female, the rate of eggs remaining per female and the total number of eggs per female were counted. The calculation of the relevant indicators is presented as follows:

*D_i_* − *A*se = *D_i_* − *T*se/*D_i_* − *T*sf (1)

*A*se = *T*se/*T*f (2)

*T*e = *T*se + *dT*e (3)

*A*re = *T*re/*T*f (4)

*R*re = *T*re/*T*e × 100% (5)

where *D_i_* − *A*se indicates the average number of self-laying eggs on day *i*; *D_i_* − *T*se is the total number of self-laying eggs on day *i*; *D_i_* − *T*sf is the number of surviving female moths on day *i*; *A*se indicates the average self-laying of eggs per female; *T*se is the total number of self-laying eggs over the lifetime; *T*f is the total number of female moths in the treatment; *T*e is the total number of *S. c. ricini* eggs; *dT*e represents the total number of eggs obtained by dissecting; *A*re indicates the average number of remaining eggs per female; *T*re indicates the number of remaining eggs; *R*re is the rate of remaining eggs.

#### 2.2.2. Developmental Duration

We collected female *S. c. ricini* within 0.5 h of emergence and divided them into three equal parts. The female moths were cultured at 25 °C for 0 h, 5 h and 24 h and then dissected to retrieve the eggs, and there were no additional nutrients during the culture period. After the eggs were washed with detergent and dried, the number of immature eggs, empty eggshells and qualified eggs were counted immediately, and the total number of qualified eggs was calculated (Equation (6)). The eggs characterized by thin eggshells, the easy loss of moisture within the egg and a greenish color were defined as immature eggs. The identification of qualified and immature eggs was made in accordance with the local standard of Guangdong Province [44].

*T*qe = *T*se + *dT*qe (6)

where *T*qe indicates the total number of qualified eggs, *T*se is the total number of self-laying eggs and *dT*qe represents the total number of qualified eggs obtained through dissection.

#### 2.2.3. Functional Response

This experiment was carried out in an artificial climatic chamber with 25 °C, RH 70 ± 5%, L:D = 14:10 to explore the functional response of *T. chilonis* to the eggs of *S. c. ricini*. The host density was set to five treatments (13, 20, 40, 80, 120 eggs), with 10 replications for each treatment. At the beginning of the experiment, suitable egg masses were selected and cut, along with the spawning paper, to eventually form similar-sized egg cards. In a flat-bottomed glass tube (2 cm in diameter and 8 cm in length), an egg card with a single layer of 13, 20, 40, 80 and 120 freshly laid eggs of *S. c. ricini* was inserted. The test female wasp was *T. chilonis* within the parasitized eggs of *C. cephalonica*. Wasp cards were prepared before the test; each wasp card contained 100 eggs of a *C. cephalonica* that was parasitized by *T. chilonis*, and the parasitized eggs were due to emerge from the *T. chilonis* adults in 2 h. According to the local standard of Guangdong Province [44], each wasp card containing 100 eggs of *C. cephalonica* parasitized by *T. chilonis* will produce about 40 adult females of *T. chilonis*. After 24 h of parasitism, the parent wasps were removed, and the parasitized eggs of *S. c. ricini* were returned to the climatic chamber to continue cultivation. Breathable gauze was used to plug the mouth of the tube, and no food was provided to *T. chilonis* during the test period. The parasitism rate (*PR*) and emergence rate (*ER*) were calculated using the following equation:

*PR* (%) = *N*p/*N*h × 100% (7)

*ER* (%) = *eN*p/*N*p × 100% (8)

where *N*p is the number of parasitized eggs and *N*h is the total number of host eggs in a treatment, while *eN*p indicates the number of parasitized eggs that emerged.

The methodology used to establish the experimental population life table for *T. chilonis* parasitized by the eggs of *S. c. ricini* was referenced in previous research [45,46], and the calculation of the life table parameters, including net reproduction rate (*R*_0_), mean generation time (*T*), intrinsic natural growth rate (*r*) and finite growth rate (λ), can be found in the work of Xu et al. [47], as described in detail in our previous study [48].

The analysis of the experiment aiming to determine the functional response of *T. chilonis* to the eggs of *S. c. ricini*, was performed in two steps [49]. Firstly, a regression analysis of the number of parasitized hosts (*N_a_*) compared to the initial host density (*N*_0_) was performed to determine the type of functional response obtained using logistic regression. The polynomial function was fitted as follows:

(9) $$\frac{N_{a}}{N_{0}}=\frac{\exp\left(P_{0}+P_{1}N_{0}+P_{2}N_{0}^{2}+P_{3}N_{0}^{3}\right)}{1+\exp\left(P_{0}+P_{1}N_{0}+P_{2}N_{0}^{2}+P_{3}N_{0}^{3}\right)}$$

where *N*_a_ is the number of hosts parasitized, *N*_0_ is the initial host density, and *P*_0_, *P*_1_, *P*_2_ and *P*_3_ are the intercept, linear, quadratic and cubic coefficients, respectively. The maximum likelihood estimation was used for parameter estimation [50]. Secondly, the type of functional response was judged according to the positive and negative parameters: a type I functional response was considered when *P*_1_ = 0, type II functional response when *P*_1_ < 0 and a type III functional response when *P*_1_ > 0 and *P*_2_ < 0 [51]. After determining the type of functional response, the handling time and instantaneous attack rates for functional response type II were assessed using Rogers’ random parasitoid model [52]:

(10) $$N_{a}=N_{0}(1-\exp(\alpha (T_{h}N_{a}-T)))$$

In which *N*_a_ is the number of hosts parasitized, *N*_0_ is the initial host density, α is the instantaneous attack rate (h^−1^), *T*_h_ is the handling time used by the parasitoid wasp to treat one host in hours and *T* is the total parasitic time (24 h).

The handling time and instantaneous attack rates for functional response type III were estimated using the following equations:

(11) $$N_{a}=ae^{-b/N_{0}}$$

*N*_a_ is the number of hosts parasitized; *a* is the maximum number of parasitized; *b* is the optimal search density; *N*_0_ is the host density.

#### 2.2.4. Wasp-to-Egg Ratios

We collected fresh parthenogenetic self-laying eggs of *S. c. ricini* laid within 24 h, which were washed with detergent and dried for the experiment. Five treatments were set up in this experiment, including *T. chilonis*-to-*S. c. ricini* egg ratios of 3:1, 2:1, 1:1, 1:2 and 1:3, with 10 replicates for each treatment. The corresponding treatments filled the tubes with 13, 20, 40, 80 and 120 *S. c. ricini* eggs (Table 1) in the form of loose eggs in the centrifuge tubes and the position of the eggs in the tubes was adjusted so that they were at the other end, away from the opening of the centrifuge tubes, and were in a single flat layer.

This experiment in this section was carried out using the above-mentioned wasp cards (2.2.3). The wasp card was inserted into the centrifuge tube and the opening of the tube was sealed with breathable paper and a rubber band. All the centrifuge tubes in the experiment were placed horizontally and flat in the light environment, making sure that the end with *S. c. ricini* eggs was towards the light and that all *S. c. ricini* eggs in the tubes were in the light. To avoid the reparasitization of *S. c. ricini* eggs by the emerged second-generation *T. chilonis*, on the 7th day of the experiment, the severely dried out *S. c. ricini* eggs that were not parasitized, the hatched *S. c. ricini* larvae that were removed from each centrifuge tube, the wasp cards and the parents of *T. chilonis* that had emerged were cleared; only the parasitized eggs of *S. c. ricini* that had turned black were retained and the mouths of the tubes were resealed with breathable paper and rubber bands. Then, the eggs were returned to the artificial climatic chamber for further development. After the offspring of *T. chilonis* had emerged and died, the number of females and males of *T. chilonis* offspring and the number of parasitized eggs that emerged from *T. chilonis* was recorded in each tube. The *S. c. ricini* eggs from which *T. chilonis* did not emerge were dissected one by one to check whether they were parasitized; then, the number of parasitized eggs from which *T. chilonis* did not emerge was recorded. The number of parasitized eggs, adult offspring and female offspring were counted, and the parasitism rate (Equation (7)), emergence rate (Equation (8)) and female sex ratio of offspring were calculated per tube as follows:

*N*p = *eN*p + *neN*p (12)

*fR*o = *NF*o/*NA*o × 100% (13)

where *N*p is the number of parasitized eggs; *eN*p indicates the number of parasitized eggs that emerged; *neN*p denotes the number of parasitized eggs that did not emerge; *fR*o is the female ratio of *T. chilonis* offspring; *NF*o represents the number of *T. chilonis* female offspring; *NA*o indicates the number of *T. chilonis* offspring adults.

### 2.3. Statistical Analysis

All data were statistically analyzed and processed using Excel 2010 (Microsoft Corporation, Redmond, WA, USA), statistical tests were performed on the computer using the software SAS V9.0 (SAS Institute Inc., Cary, NC, USA) and the NLIN program in SAS was used to estimate the parameters of attack rate (α) and handling time (Th). Significant differences among treatments were determined as one-way ANOVA and Duncan’s new complex polar difference method was used for multiple comparisons. *S. c. ricini* female survival rate was compared through a Kaplan–Meier survival analysis followed by a log rank test in GraphPad Prism 8 software (GraphPad Software Inc., San Diego, CA, USA). The graphs were drawn using Excel 2010 and GraphPad Prism 8 software.

## 3. Results

### 3.1. Effects of Developmental Temperature on the Longevity and Oviposition of S. c. ricini

**Figure 1:**
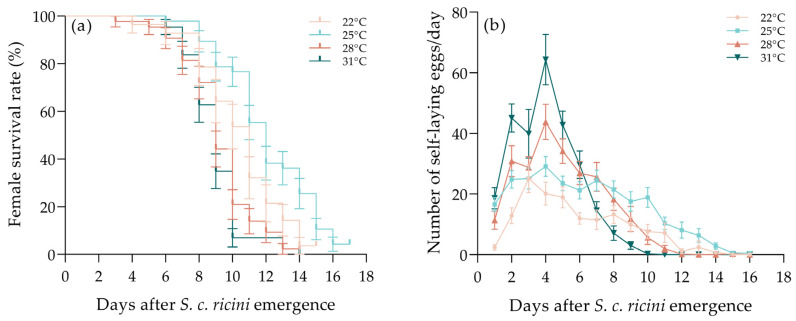
Daily changes in survival rate (**a**) and the number of self-laying eggs of *S. c. ricini* females (**b**) at different temperatures.

There are some differences in the longevity and oviposition of female adults when using different developmental temperatures. The survival rate of female adults per day, at 25 °C, was higher than that of the other treatments, and the lowest was observed at 31 °C (Figure 1a); however, the difference in survival rate between these treatments was not significant (χ^2^ = 3.6, df = 3, *p* = 0.3). The longevity of female moths, at 25 °C, was significantly longer than the other treatments (Table 2), and the longevity at 28 °C and 31 °C was significantly shorter than that at 22 °C and 25 °C (F = 16.299, df = 3, *p* < 0.001). Females of *S. c. ricini* were observed to have an oviposition period of about 15 days and the average number of self-laying eggs per day showed an upward and then downward trend (Figure 1b), which reached a peak at 3–5 days and then declined each day. The peak period of oviposition was within 1 week after the emergence. The average number of self-laying eggs per day increased with temperature within 1–6 days after the emergence of female adults. At the 22 °C culture conditions, the total number of self-laying eggs of *S. c. ricini* was significantly lower than the other treatments (F = 9.559, df = 3, *p* < 0.001), whereas the average number of remaining eggs (F = 6.414, df = 3, *p* < 0.001) and the rate of remaining eggs (F = 6.418, df = 3, *p* < 0.001) were considerably higher than in the other treatments. The total number of eggs of *S. c. ricini* was significantly higher at 28 °C and 31 °C than at 22 °C (F = 3.195, df = 3, *p* < 0.001.025), whereas the differences in the total number of self-laying eggs and the average number of remaining eggs per female, the rate of remaining eggs and the total number of *S. c. ricini* eggs were not significant among the treatments of 25 °C, 28 °C and 31 °C.

### 3.2. Effects of Developmental Duration on the Quality of S. c. ricini Eggs

**Figure 2:**
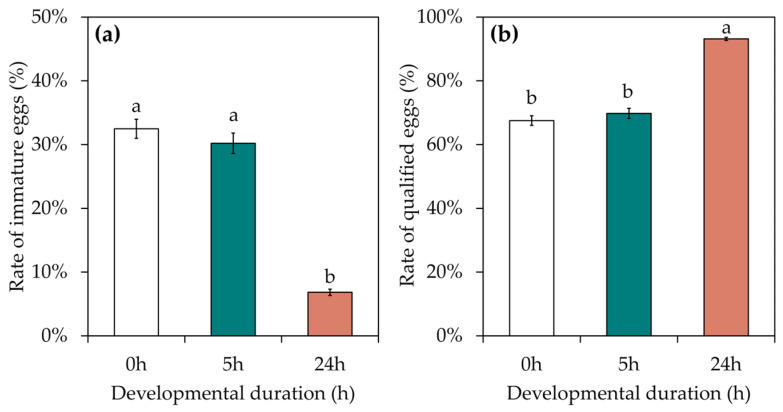
Effects of developmental duration on the egg quality of *S. c. ricini*. (**a**) The rate of immature eggs (%); (**b**) the rate of qualified eggs (%). The columns in the figure represent the average value for that treatment, the error bars indicate the standard error and different lowercase letters in the figures indicate significant differences at the *p* < 0.05 level, as tested by Duncan’s new complex polarity test.

The female moths of *S. c. ricini* were collected just after emergence. Of the eggs obtained via immediate dissection, the rates of immature and qualified eggs were 32.48 ± 1.87% and 67.52 ± 1.87% (Figure 2), respectively. After the development of the female moths at 25 °C for 5 h, the rates of immature and qualified eggs received from dissections were 30.21 ± 5.20% and 69.79 ± 5.20%, respectively, and there was no significant increase in the rate of qualified eggs compared with the development of 0 h. Among the female moths that continued to develop until 24 h and then were dissected for eggs the rate of immature eggs (6.83 ± 1.52%) was significantly lower than that obtained using the other treatments (F = 18.407, df = 2, *p* < 0.001), whereas the rate of qualified eggs (93.17 ± 1.52%) was significantly higher than in the other treatments (F = 18.407, df = 2, *p* < 0.001). After 24 h of development, the eggs of *S. c. ricini* matured and were successively produced. To avoid the high rate of immature eggs obtained through dissection, it is necessary to give the female moth sufficient development time to obtain a higher rate of qualified eggs.

### 3.3. Functional Response of T. chilonis to S. c. ricini Eggs

#### 3.3.1. Population Growth of *T. chilonis*

The egg density of *S. c. ricini* has a significant effect on the population growth of *T. chilonis* (Table 3). The average total number of *T. chilonis* (*TW*) and the average number of *T. chilonis* females (*FW*) produced by *S. c. ricini* eggs were significantly higher at egg densities of 80 and 120 than at densities of 13, 20 and 40, and at the egg density at 40, the results were also significantly higher than those obtained at a density of 13. With the increase in host density, the average number of *T. chilonis* females produced by a single *S. c. ricini* female (*FW*_pf_) also increased. At egg densities of 80 and 120, the net reproductive rate of *T. chilonis* was found to be significantly higher than that of other treatments (*p* < 0.05). Moreover, the intrinsic rate of increase and the finite rate of increase of *T. chilonis* exhibited a significant upward trend with the increase in egg density. Except for the treatment with an egg density of 13, the population multiplication time of *T. chilonis* was significantly shorter with increasing egg density. These results suggest that a host egg density of 120 was most favorable for population reproduction within the density range of this experiment.

#### 3.3.2. Parasitic capacity of *T. chilonis*

**Figure 3:**
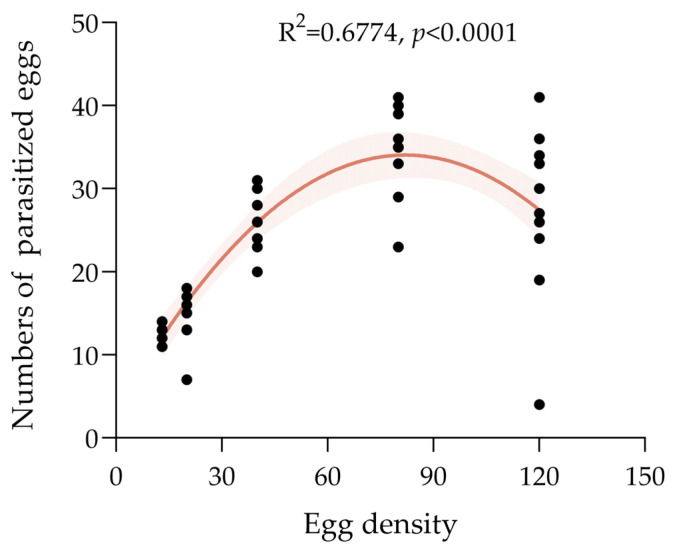
Functional response of *T. chilonis* to *S. c. ricini* eggs over 24 h period; the light orange sections represent 95% confidence intervals.

A logistic regression analysis showed the linear coefficient of *P*_1_ < 0 for *S. c. ricini* eggs parasitized by *T. chilonis*, which indicates that the functional response was type II (Table 4). The Holling type II model was further fitted to obtain the functional response model for each temperature condition (Table 5). The instantaneous attack rate (α) of the *T. chilonis* parasitizing *S. c. ricini* eggs was 0.1840 and the handling time (*T*_h_) was 0.4628 d. The maximal parasitic was 2.16 eggs and the *r* of the functional equation was 0.9703, which provided a good fit to the equation. The parasitism of *T. chilonis* on the eggs of *S. c. ricini* increased with host density to a certain amount and then stopped, and the trend line equation showed a good match (Figure 3).

### 3.4. Effects of the Wasp–Egg Ratio on the Reproduction of T. chilonis Using S. c. ricini Eggs

**Figure 4:**
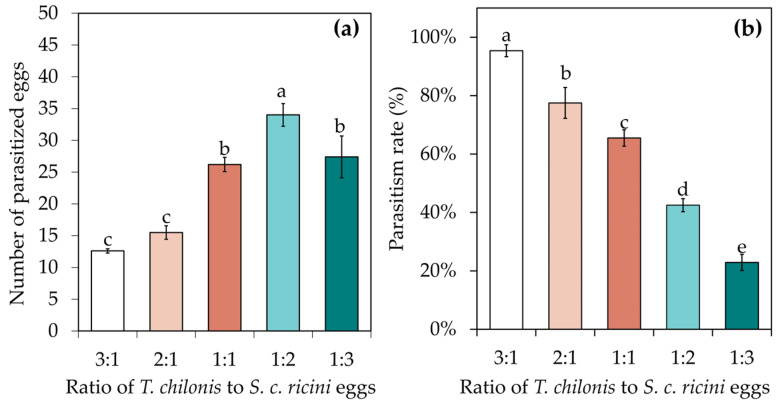
Effects of the wasp–egg ratio on the parasitism efficacy of *T. chilonis* on *S. c. ricini* eggs. (**a**) The number of parasitizing eggs; (**b**) parasitism rate (%). The columns in the figure represent the average value for that treatment, the error bars indicate the standard error and different lowercase letters in the figures indicate significant differences at the *p* < 0.05 level, as tested by Duncan’s new complex polarity test.

The wasp-to-egg ratio had a significant effect on the parasitism efficacy of *T. chilonis* (Figure 4). The number of parasitized eggs increased and then decreased as the wasp-to-egg ratio grew smaller, and the treatment with a wasp-to-egg ratio of 1:2 led to the highest number of parasitized eggs (34.00 ± 1.79), with significantly more eggs than that of the other four treatments. However, the number of parasitized eggs obtained using wasp-to-egg ratios of 3:1 and 2:1 was significantly lower than that obtained with the other three treatments (F = 23.859, df = 4, *p* < 0.001) (Figure 4a). The parasitism rates varied significantly among treatments; the lower the number of *S. c. ricini* eggs, the higher the parasitism rate for the same number of *T. chilonis* (Figure 4b).

In addition, there was a significant effect of wasp-to-egg ratio on the survival and development of *T. chilonis* offspring (Table 6). After parasitizing the eggs of *S. c. ricini*, *T. chilonis* offspring develops and lives inside the eggs until it emerges, bites through the shell of the parasitized eggs and drills its way out. The treatments with wasp-to-egg ratios of 1:2 and 1:3 had significantly more parasitized eggs that emerged (*eN*p) than the other three treatments (*F* = 19.47, df = 4, *p* < 0.001). The emergence rate (*ER*) increased with the number of *S. c. ricini* eggs. The emergence rate of the treatment with a wasp-to-egg ratio of 3:1 was notably lower than that in the other treatments, whereas that in the treatment with a wasp-to-egg ratio of 1:3 was higher than that in the other four treatments (*F* = 13.816, df = 4, *p* < 0.001). Additionally, the number of *T. chilonis* in the parasitized eggs that did not emerge (*neN*w) in the treatments with wasp-to-egg ratios of 1:2 and 1:3 was significantly lower than in the treatments with ratios of 3:1 and 2:1 (*F* = 8.823, df = 4, *p* < 0.001). Moreover, the number of adults (*NA*o, *F* = 15.954, d = 4, *p* < 0.001) and female adults of *T. chilonis* offspring (*fNA*o, *F* = 16.127, d = 4, *p* < 0.001), and the propagation coefficient (*PC*, *F* = 16.127, df = 4, *p* < 0.001), in the treatments with wasp-to-egg ratios of 1:2 and 1:3 were considerably higher than in the other treatments; all five treatments showed a high female ratio of over 85% in the *T. chilonis* offspring. When *S. c. ricini* eggs are used at a wasp-to-egg of 1:2–1:3, the same number of *T. chilonis* female parents can breed a higher number of adult offspring and female offspring, which exhibit an increased reproductive efficiency.

## 4. Discussion

**Figure 5:**
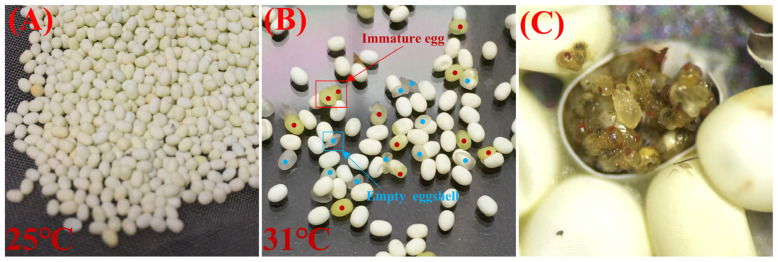
Morphology of eggs laid by *S. c. ricini* female adults incubated at 25 °C (**A**) and 31 °C (**B**), and developmental status of *T. chilonis* in *S. c. ricini* eggs (**C**). The red dots in the figure indicate immature eggs and the blue dots represent empty eggshells.

Suitable intermediate host eggs are the key to breeding *Trichogramma* parasitoids, because the quality of the host eggs is related to the availability of nutrients for development, affecting the parasitism and emergence rates. The use of suitable host eggs also determines the propagation efficiency and cost of breeding *Trichogramma* parasitoids, while the knowledge of the intermediate host’s living characteristics and response to changes in the external environment provides the necessary basis to carry out the mass rearing of intermediate hosts and quality controls [9]. Insects are typical thermotropic animals, and temperature is an important environmental factor that affects their growth, development, reproduction and behavioral activities [53,54,55]. Previous studies have concluded that *S. c. ricini* is a non-stagnant insect; the appropriate temperature for indoor culture is 20–28 °C, and the longevity of adults is 10–12 d [34,56,57]. The results observed in this study are basically consistent with these findings. Our results suggest that post-feathered *S. c. ricini* have a higher survival rate and longer lifespan at 25 °C compared to that at lower (22 °C) and higher (28, 31 °C) temperatures, suggesting that the appropriate survival temperature for adults under laboratory conditions is 25 °C. Similarly, Wongsorn et al. [57] found that *S. c. ricini* survival rates and total egg production had maximum values of 25 °C compared to other temperature treatments. Generally, the developmental rate of insects starts at the critical thermal minimum and rises slowly with increases in temperature. The development rate rises almost linearly over a range of temperatures, continues to rise to the optimum level and finally falls rapidly following the critical maximum temperature [58]. Higher ambient temperatures can accelerate senescence by promoting insect development, shorten lifespan and worsen infection-based outcomes [59]. In addition, temperature significantly affected the oviposition of *S. c. ricini*. At lower temperatures (22 °C), *S. c. ricini* had fewer self-laying eggs and had a higher number of remaining eggs. Previous studies on several species of moths have indicated that egg production tended to increase and then decrease with temperature increases, i.e., egg production reached a maximum at a certain temperature, and excessively high or low temperatures lead to a decrease in egg production [60]. We found that the total number of *S. c. ricini* eggs increased with temperature in the range of 22 °C to 31 °C, whereas Wongsorn et al. [57] observed that in the temperature range of 25 °C to 48 °C, the total number of *S. c. ricini* eggs peaked at 36 °C (300.53 eggs/moth) and then declined. However, it is notable that we observed some abnormalities in the color of self-laying eggs at 31 °C during the experiment, and the appearance of the eggs (flattened and rounded) was not as good as that of the eggs formed under the treatments of 25 °C and 28 °C (Figure 5 presents the differences in the appearance of eggs; quantitative data of viable eggs were not counted in this study). Prior research has suggested that high temperatures (29 °C and 32 °C) may have an impact on egg quality by affecting ovarian development [61], but it has also been argued that high-temperature heatwaves (5–7 °C above optimal temperature) primarily influence male fecundity and sperm competitiveness, without impairing female reproduction [62]. In addition to the significant effect of temperature on the longevity and oviposition of *S. c. ricini*, the developmental duration after emergence also has a significant effect on oviposition. Normally, many insects require a period of time after emergence to achieve sexual maturity [63], and understanding the time it takes for insects to reach physiological maturity could help to deepen our knowledge. We found that more unqualified eggs were obtained at 0 h after emergence, whereas more qualified *S. c. ricini* eggs could be obtained 24 h after emergence. Harris and Rose [35] found that the development duration after emergence affected the time for *Mayetiola destructor* (Say) to reach sexual maturity, which in turn determined the onset of egg laying and affected the production of eggs and the distribution of egg laying time. For example, for females that mated 1, 2 and 3 h after emergence, the durations of the post-mating pre-ovipositional transition phase were 190, 160 and 120 min, respectively [35]. A sufficient developmental duration ensures the normal development and refinement of the insect’s reproductive organs so that the insect can lay eggs normally. If development is incomplete, there may be difficulty in laying eggs or a decrease in egg production. In summary, we concluded that 25–28 °C is the appropriate temperature range for the survival and egg-laying of *S. c. ricini* adults, and that adults need to experience at least 24 h of development after emergence to lay more qualified eggs.

Functional response analyses are often used to help predict the potential of parasitoids to regulate host populations [64,65]. Our results indicated that *S. c. ricini* exhibits a type II functional response to *S. c. ricini* eggs; the average number of hosts that parasitized eggs gradually increased with host density and attained the upper asymptote at an egg density of 80. Type II functional responses were common in *Trichogramma* spp.; more than 75% of relevant studies reported a type II functional response [42], and past studies have reported that *T. chilonis* showed type II functional responses to *T. absoluta* [42], *C. cephalonica* [7] and *C. sacchariphagus* [43]. The handling time of a Type II functional response involves subduing the host, accepting the host, spawning and then possibly cleaning and resting before moving on to find more hosts. Type II functional responses described an inverse density-dependent relationship between the proportion of parasitoids and the density of the hosts [36], i.e., biological agents are more effective at low pest densities. This provides an insight into the best means of field release for biological agents. Further references are provided for indoor *T. chilonis* reproduction, that is, low-density intermediate host eggs might be able to obtain a higher parasitism rate, which could lead to cost reductions and an increase in efficiency. For this reason, we carried out experiments on the reproduction of *T. chilonis* at different wasp–egg ratios, and our results showed that the parasitism rate decreased significantly with an increase in the number of *S. c. ricini* eggs when using the same number of *T. chilonis*. A similar phenomenon was observed by Li et al. [66] regarding the use of *C. cephalonica* eggs for *T. chilonis* reproduction. In addition, we found that the number of parasitized eggs that emerged, the emergence rate, the number of *T. chilonis* that emerged per *S. c. ricini* egg and the total number of *T. chilonis* gradually increased with the number of *S. c. ricini* eggs, which was consistent with the results observed by Lu et al. [67] when using *A. pernyi* eggs to breed *T. chilonis*. Compared to small eggs (*C. cephalonica* and *S. cerealella*), large eggs (*A. pernyi* and *S. c. ricini*) may contain more and richer nutrients, such as proteins and fats, providing more sufficient nutrients for the growth and development of *T. chilonis* larvae, which could lead to the production of more wasps. We observed the highest number of *T. chilonis* (186.80 ± 22.73) produced by a single *S. c. ricini* egg in the treatment with wasp–egg ratio of 1:3. However, not every parasitized egg successfully emerged, and previous studies have shown that some *Trichogramma* spp. can develop into adults in *A. pernyi* eggs but ultimately die in the egg because they are unable to bite through the egg shell [23,68]; this may be related to the lower emergence rates we observed. Furthermore, with an increase in proportion of the inoculation of parasitoids and host eggs, the frequency of superparasitism increased, and the body size, sex ratio of female offspring and number of emerged adults reduced [69]. This might explain the few emergences found in the wasp-to-egg treatments of 3:1 and 2:1 in our study. There was no significant difference in propagation performance between the treatments with wasp-to-egg ratios of 1:2 and 1:3, but there was a significant difference in emergence rate; therefore, based on the cost of *S. c. ricini* egg supply, a wasp-to-egg ratio of 1:2 may be suitable for *T. chilonis* propagation.

Although we explored the effects of developmental temperature and duration on the development and oviposition of *S. c. ricini* adults, other conditions, such as light, humidity and food sources, are also important influencing factors. In addition, egg management and quality control techniques, such as egg acquisition methods and egg storage, are important factors affecting the propagation efficacy of *T. chilonis*. Therefore, in the future, we should strengthen the knowledge of some important biotic and abiotic factors affecting the production of castor silkworms to provide a scientific basis for optimizing the breeding environment of *S. c. ricini* and improving the production of *S. c. ricini*, as well as to provide technological support for the establishment of a complete system of *S. c. ricini* egg production, storage and usage.

## 5. Conclusions

Our results indicated that developmental temperature and duration after the emergence of *S. c. ricini* significantly affected the survival and oviposition of adults, that a suitable temperature range for the survival and egg-laying of adults after emergence is 25–28 °C, and that at least 24 h of development is required to obtain more qualified *S. c. ricini* eggs. *T. chilonis* exhibited a type II functional response to *S. c. ricini* eggs. The usage of *S. c. ricini* eggs for the reproduction of *T. chilonis* resulted in a high female ratio among offspring and *S. c. ricini* eggs showed an excellent reproductive performance. Different ratios of *T. chilonis* to *S. c. ricini* eggs significantly influenced the reproduction performance, with more *T. chilonis* offspring per *S. c. ricini* egg being obtained under the treatments with wasp-to-egg ratios of 1:2 and 1:3, showing a high propagation efficiency. However, given the cost of eggs, we concluded that a wasp-to-egg ratio of 1:2 is suitable for the reproduction of *T. chilonis*. There are still more aspects to be explored regarding the use of *S. c. ricini* for the reproduction of *T. chilonis*; however, our results may provide some valuable insights into the reproduction of *T. chilonis*.

## Figures and Tables

**Table 1:** Number of *T. chilonis* and *S. c. ricini* eggs used in different treatments.

Treatments (Wasp-to-Egg Ratio)	3:1	2:1	1:1	1:2	1:3
Number of *T. chilonis*	39	40	40	40	40
Number of *S. c. ricini* eggs	13	20	40	80	120

**Table 2:** The average longevity and eggs number of *S. c. ricini* females under different temperatures.

Temperature (°C)	Longevity (d)	*T*se	*A*re	*R*re (%)	*T*e
22	10.50 ± 0.49 _b_	144.4 ± 16.2 _b_	95.2 ± 17.5 _a_	35.81± 6.39 _a_	239.6 ± 17.9 _b_
25	12.13 ± 0.40 _a_	251.4 ± 13.5 _a_	27.2 ± 7.2 _b_	11.89 ± 3.27 _b_	273.3 ± 11.5 _ab_
28	9.28 ± 0.34 _c_	239.6 ± 14.9 _a_	42.3 ± 10.8 _b_	15.08 ± 3.47 _b_	281.9 ± 12.2 _a_
31	9.02 ± 0.26 _c_	265.1 ± 17.5 _a_	39.5 ± 9.2 _b_	14.02 ± 3.24 _b_	304.6 ± 14.9 _a_

Note: *T*se is the total number of self-laying eggs over the lifetime; *A*re indicates the average number of remaining eggs per female; *R*re is the rate of remaining eggs; *T*e is the total number of *S. c. ricini* eggs. Data in the table are the average ± standard errors, and different lowercase letters in the same column indicate significant differences at the *p* < 0.05 level, as tested by Duncan’s new complex polarity test.

**Table 3:** Effects of egg density of *S. c. ricini* on the life table parameters of the experimental population of *T. chilonis*.

Egg Density	*R*_0_	*r_m_*	*λ*	*T* (*d*)	*FW*_pf_	Sex Ratio (%)	*FW*	*TW*
13	0.475 ± 0.028 ^c^	−0.073 ± 0.032 ^e^	0.930 ± 0.010 ^c^	−9.50 ± 0.17 ^d^	0.485 ± 0.271 ^e^	97.9 ± 2.74 ^a^	19.0 ± 6.88 ^c^	19.4 ± 7.07 ^c^
20	1.088 ± 0.318 ^b^	0.008 ± 0.027 ^d^	1.008 ± 0.018 ^b^	83.51 ± 0.89 ^a^	1.238 ± 0.885 ^d^	87.8 ± 4.95 ^b^	43.4 ± 16.15 ^bc^	49.4 ± 18.11 ^bc^
40	1.893 ± 0.265 ^b^	0.063 ± 0.006 ^c^	1.063 ± 0.027 ^b^	11.07 ± 0.37 ^b^	2.047 ± 0.713 ^c^	92.4 ± 2.02 ^ab^	75.7 ± 12.43 ^b^	81.9 ± 14.40 ^b^
80	4.033 ± 1.893 ^a^	0.137 ± 0.012 ^b^	1.146 ± 0.008 ^b^	5.07 ± 0.62 ^c^	4.280 ± 0.830 ^b^	94.2 ± 2.79 ^ab^	161.3 ± 26.38 ^a^	171.2 ± 26.78 ^a^
120	4.670 ± 1.438 ^a^	0.151 ± 0.006 ^a^	1.163 ± 0.012 ^a^	4.59 ± 0.54 ^c^	5.160 ± 1.375 ^a^	90.5 ± 1.52 ^ab^	186.8 ± 22.73 ^a^	206.4 ± 26.54 ^a^

Note: *R*_0_ is net reproductive rate; *r_m_* denotes intrinsic rate of increase; *λ* indicates finite rate of increase; *T* represents population multiplication time; *FW*_pf_ refers to the average number of female wasps (*T. chilonis*) produced per female insect (*S. c. ricini*); *FW* is the average number of female wasps (*T. chilonis*); *TW* means the average total number of wasps (*T. chilonis*). Data in the table are the means average ± standard errors; different letter in the columns represented significant difference (*p* < 0.05, Tukey’s test).

**Table 4:** Statistical parameters of logistic regression analysis.

Parameters	Estimate	Standard Error	χ^2^	*p*-Value
*P*_0_	3.4196	0.4753	51.76	<0.0001
*P*_1_	−0.1126	0.0267	17.85	<0.0001
*P*_2_	0.00125	0.000415	9.05	0.0026
*P*_3_	−0.000000528	0.0000001896	7.74	0.0054

**Table 5:** Functional response parameters of *T. chilonis*.

Functional Response Type	II-Type
Maximal parasitic (egg/d)	2.16
*A*	0.1840 ± 0.0711
*T*_h_ (d)	0.4628 ± 0.0502
Functional equation	*N*_a_ = 0.1840*N*/(1 + 0.4628*N*)
*R* ^2^	0.9703

Note: Data in the table are the average ± standard errors. α, instantaneous attack rate; *T*_h_, handling time (d).

**Table 6:** Effects of different wasp-to-egg ratios on the development and survival of *T. chilonis* offspring and reproductive efficiency.

Treatment	Development and Survival of *T. chilonis* Offspring				Reproductive Efficiency			
	*eN*p	*ER* (%)	*eN*fw	*neN*w	*NA*o	*fNA*o	*fR*o (%)	*PC*
3:1	1.20 ± 0.42 ^c^	9.39 ± 3.21 ^d^	19.00 ± 6.88 ^c^	30.24 ± 1.31 ^b^	19.40 ± 7.07 ^c^	19.00 ± 6.88 ^c^	98.41 ± 1.12 ^a^	0.4750 ± 0.1720 ^c^
2:1	3.10 ± 0.90 ^bc^	20.60 ± 5.31 ^c^	43.40 ± 16.15 ^bc^	34.55 ± 1.30 ^a^	49.40 ± 18.11 ^bc^	43.40 ± 16.15 ^bc^	85.64 ± 4.95 ^c^	1.0850 ± 0.4038 ^bc^
1:1	6.20 ± 1.04 ^b^	23.96 ± 3.96 ^c^	75.70 ± 12.43 ^b^	28.19 ± 1.19 ^bc^	81.90 ± 14.40 ^b^	75.70 ± 12.43 ^b^	94.33 ± 2.02 ^ab^	1.8925 ± 0.3107 ^b^
1:2	12.10 ± 1.63 ^a^	35.28 ± 3.87 ^b^	161.30 ± 26.38 ^a^	24.92 ± 1.28 ^c^	171.20 ± 26.78 ^a^	161.30 ± 26.38 ^a^	92.79 ± 2.79 ^ab^	4.0325 ± 0.6595 ^a^
1:3	12.60 ± 1.46 ^a^	46.87 ± 2.31 ^a^	186.80 ± 22.73 ^a^	28.87 ± 0.72 ^c^	206.40 ± 20.54 ^a^	186.80 ± 22.72 ^a^	91.83 ± 1.52 ^ab^	4.6700 ± 0.5682 ^a^

Note: e*N*p, the number of parasitized eggs that emerged; *ER*, emergence rate; *eN*fw, the number of female waps (*T. chilonis*) that emerged per egg (*S. c. ricini*); *neN*w, the number of wasps that did not emerge per egg (*S. c. ricini*); *NA*o, the number of offspring adults; *fNA*o, the number of females in offspring adults; *f*Ro, the female ratio of offspring adults; PC, propagation coefficient. Data in the table are the means average ± standard errors; different letters in the columns represent significant difference (*p* < 0.05, Tukey’s test).

## Data Availability

The data presented in this study are available from the corresponding author upon request.
